# Diagnostic Challenge in a Symptomatic Patient of Arteria Lusoria with Retro-esophageal Right Subclavian Artery and Absent Brachiocephalic Trunk

**DOI:** 10.7759/cureus.7029

**Published:** 2020-02-18

**Authors:** Hina Akbar, Arslan Kahloon, Rehan Kahloon

**Affiliations:** 1 Internal Medicine and Hospital Medicine, The University of Tennessee Health Science Center (UTHSC), Memphis, USA; 2 Gastroenterology, Erlanger Health System/UT College of Medicine, Chattanooga, USA; 3 Cardiology, Erlanger Health System/UT College of Medicine, Chattanooga, USA

**Keywords:** arteria lusoria, coronary angiography, absent right brachiocephalic, anamolous left circumflex artery, right radial artery access

## Abstract

A combination of absent brachiocepahlic trunk and anomalous left circumflex artery with a retro-esophageal right subclavian artery is an extremely rare finding. This can clinically manifest as episodic dysphagia and chest pain. Routine coronary angiography via femoral access could be misleading and right radial access in such cases can be particularly challenging and has never been reported in literature before.

We present a case of a 42-year-old female with symptoms of chest, back, and left neck pain. She also complained of occasional dysphagia, dizziness, and palpitations. Physical examination revealed a regular heart rhythm with no vascular bruits. An electrocardiogram (EKG) only showed normal sinus rhythm and incomplete right bundle branch block. Noninvasive testing included an echocardiogram and previously done exercise stress test, and myocardial perfusion scans were noted to be normal. A diagnostic cardiac catheterization via right radial approach was performed to delineate her coronary anatomy and rule out ischemic etiology. This led to diagnosis of anomalous coronary anatomy (retro-esophageal right subclavian artery arising from descending aorta in association with an anomalous right circumflex artery with absent innominate artery) through a technically difficult and risky procedure. Significant vessel tortuosity and abnormal catheter angulations were encountered and were overcome by using specific catheters. Meticulous use of 6 French MP, WR, JL, and JR4 catheters along with an exchange length wire was required to negotiate the anatomical variations and complete the coronary angiogram via right radial artery.

From a procedural stand-point, coronary angiography via right radial access in presence of such rare anatomical variations can be particularly challenging. Routine femoral catheterization may fail to depict this important anatomical variation.

Coronary angiogram via right radial access in the presence of a combination of anatomical variations of great vessels and anomalous coronary arteries is particularly challenging from a procedural stand point due to vessel tortuosity and shape of catheters. Choice of anatomically appropriate diagnostic catheters and specific maneuvers are imperative in these coronary angiographic procedures.

## Introduction

There are several types of aortic arch anomalies that can develop during gestation as a result of failure in the congenital development of the primordial aortic arch [1]. Several of these has been reported in literature including aberrant right subclavian artery (ARSA) or arteria lusoria which is the most common aortic arch anomaly with prevalence of 0.5%-2.5% of individuals [2-4]. Most of such patients do not have active symptoms. However depending on the anomaly, these anatomical variations can clinically manifest as gastrointestinal (GI) symptoms including episodic dysphagia and regurgitation, cardiac symptoms including atypical chest pain or angina, and respiratory symptoms like chronic cough, aspiration, and bronchiectasis [5-8].

A combination of such multiple aortic arch anomalies can occur together as well but is extremely rare [4]. One such variant anatomy is absent brachiocepahlic trunk along with anomalous left circumflex artery with a retro-esophageal right subclavian artery. This particular anomaly is an extremely rare finding [3-4] with variable presenting symptoms and poses a significant diagnostic dilemma. 

We report one such case where eventual diagnosis was made through complex coronary angiography via right radial access. This kind of procedure can be particularly challenging and has never been reported in the literature before. 

## Case presentation

A 42-year-old female presented to the outpatient cardiology clinic for symptoms of chest pain. The patient complained of increasing episodes of chest pain radiating towards her back and neck along with having episodes of palpitations, dizziness, intermittent dysphagia, and recurrent headaches with near syncope. Physical examination revealed a regular heart rhythm with no vascular bruits. An exercise stress test taken a year ago was normal. An echocardiogram was also normal. An electrocardiogram (EKG) revealed normal sinus rhythm and incomplete right bundle branch block. Several non invasive studies for work up of chest pain including exercise stress test and myocardial perfusion test were negative. She remained symptomatic with a growing concern of underlying coronary artery disease and was subsequently scheduled for a diagnostic cardiac catheterization to delineate her coronary anatomy. 

**Figure 1:**
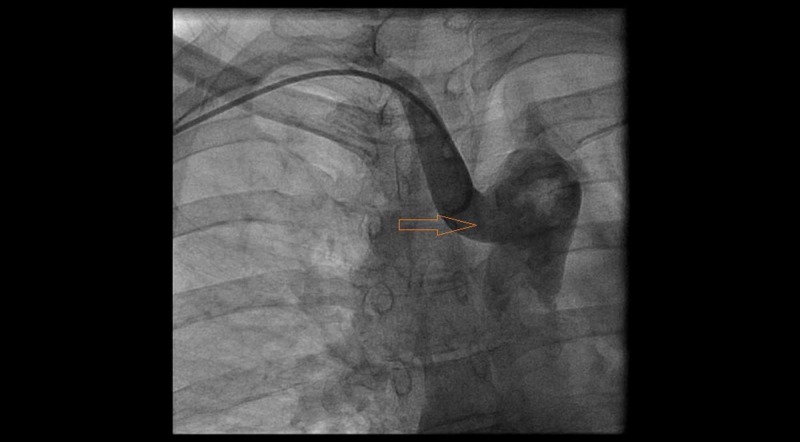
Nonselective aortic arch injection showing aberrant retro-esophageal right subclavian artery originating from descending aorta.

The patient was prepped and draped on catheterization table in a sterile fashion and right radial artery was accessed under ultrasound guidance. After insertion of a 6 French slender radial sheath a 0.035 J tipped was advanced but it failed to advance in aortic arch and ascending aorta. Instead wire kept advancing easily in descending aorta. After multiple failed attempts we decided to perform a nonselective aortic arch injection (Figure 1).

**Figure 2:**
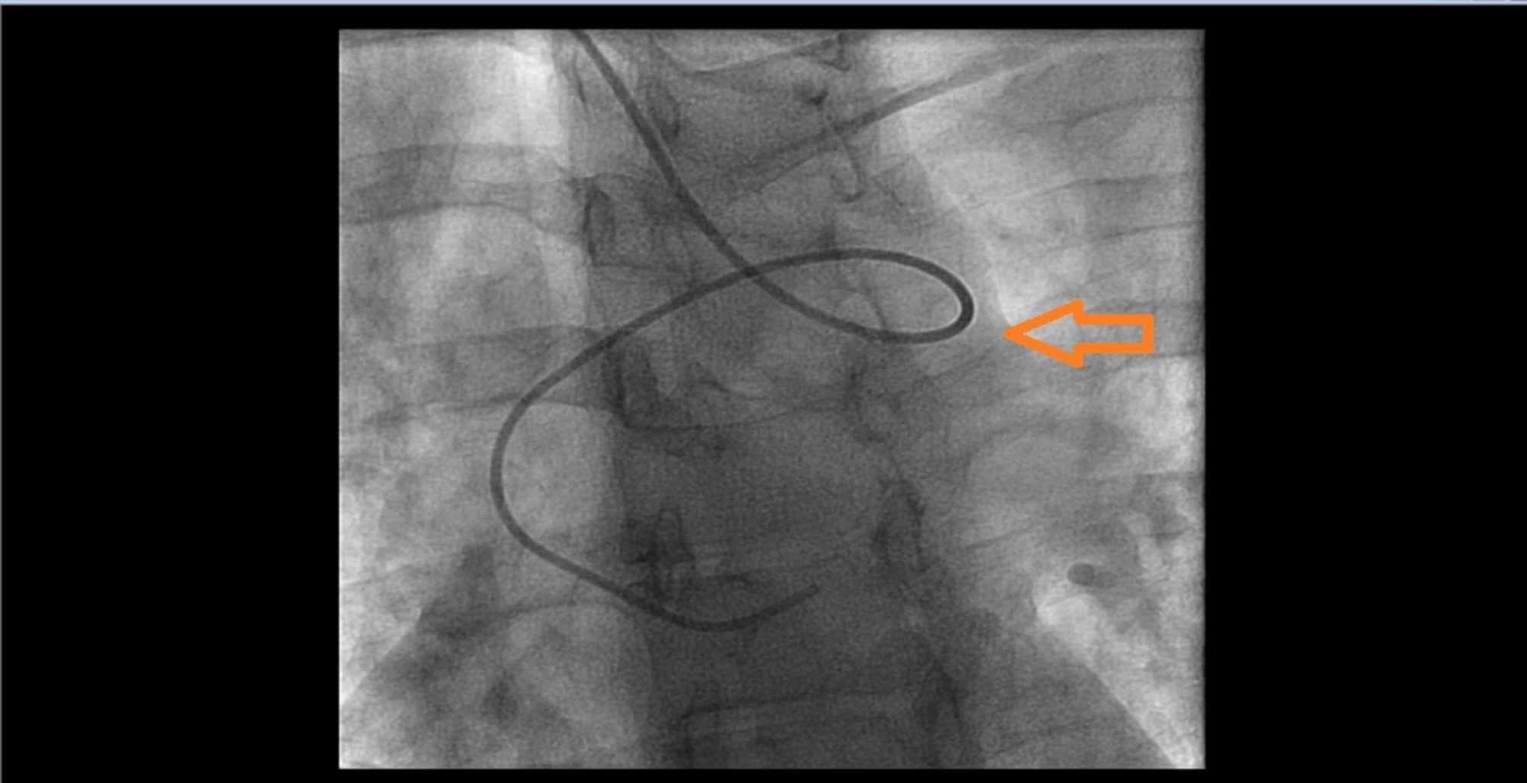
6 French Judkins Left 4.0 diagnostic catheter engaging left main coronary ostium with significant tortuosity of the aberrant right subclavian artery.

**Figure 3:**
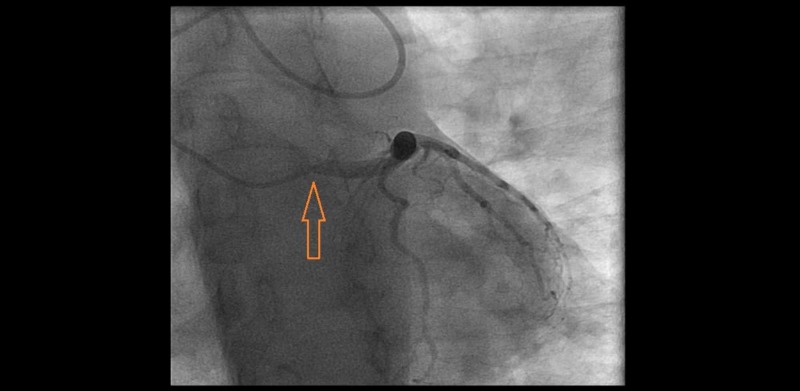
6 French Judkins Left 4.0 catheter engaging left main artery.

**Figure 4:**
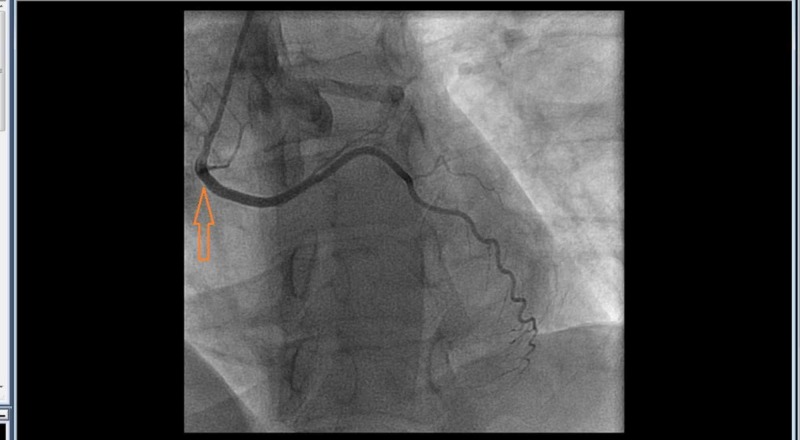
Anomalous circumflex artery originating from right coronary ostium. 6 French multipurpose catheter engaging the circumflex artery

Injection revealed an aberrant right subclavian artery originating from the descending aorta. Given significant tortuosity (Figure 2) and risk of complications with further advancing the catheter, we contemplated to abort radial access and move to femoral access. However, we then meticulously used a 6 French Multi Purpose catheter, a 6 French Williams Right catheter , a 6 French Judkins Left 4.0, and a 6 French Judkins Right 4 catheters along with a long exchange length 0.035 J tipped wire to negotiate the anatomical variations and completed the selective coronary angiograms successfully (Figures 3-4). 

Once the anomalous anatomy was confirmed and no structural ischemic etiology was noted, the procedure was completed without any complications. 

**Figure 5:**
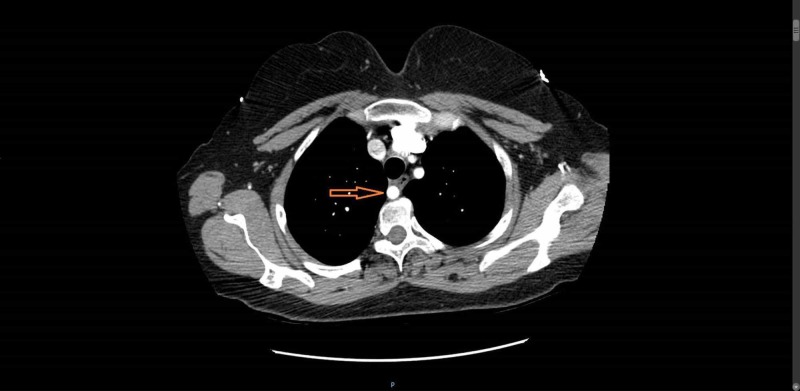
Chest CT scan showing retro-esophageal aberrant right subclavian artery.

The patient later underwent a CT scan of chest with contrast which confirmed the anomalous anatomy that was also seen seen during catheterization (Figure 5). 

The patient's symptoms of atypical chest pain and intermittent dysphagia were thought to be related to this anomalous anatomy. The patient was offered both surgical evaluation and medical therapy with beta blockers, calcium channel blockers, and GI anti-spasmodics (hycosamine). She opted for medical therapy and her symptoms responded very well to medical management.

## Discussion

The presence of a retro-esophageal right subclavian artery arising from descending aorta in association with an anomalous right circumflex artery with absent innominate artery is an extremely rare finding when found as a combination [2-4]. This variant of aortic arch anomalies can pose a diagnostic challenge due to nonspecific symptom presentation leading to routine invasive procedure which may end up being technically difficult and risky intra-operatively [3-7]. Routine femoral catheterization may fail to depict this important anatomical variation. Coronary angiogram via right radial access can be particularly challenging from a procedural stand-point as significant vessel tortuosity and abnormal catheter angulations may be encountered unexpectedly [4]. Nonselective aortic arch injection may help delineate the anatomy. A 6 French mutipurpose catheter can still be employed to engage the anomalous circumflex artery originating from the right coronary cusp. 

We hereby report our unique technique using particular catheters and guidewires to help for successful completion of this otherwise difficult procedure. Currently no guidelines or expert opinions exist in the literature to perform coronary angiogram via right radial access in these anatomic variations. Our findings were also confirmed on a contrasted chest CT subsequently which can be helpful if available pre-operatively to plan the procedure accordingly.

Patients having episodic dysphagia and chest pain with negative GI and noninvasive cardiovascular work up should be investigated for the presence of retro-esophageal aberrant right subclavian artery and other coronary anomalies . Chest CT with contrast can be an important diagnostic modality in the work up of these patients. As encountered in our case for above mentioned reasons, a coronary angiogram and left heart catheterization from right radial access can prove to be technically challenging and risky. In these patients left radial artery access or femoral arterial approach can potentially result in shorter procedure and sedation times with less risk of complications. 

## Conclusions

Left heart catheterization and coronary angiogram via right radial access in the presence of a combination of anatomical variations of great vessels and anomalous coronary arteries is particularly challenging from a procedural stand-point as significant vessel tortuosity and abnormal catheter angulations may be encountered. Choice of anatomically appropriate diagnostic catheters and specific maneuvers are imperative in these coronary angiographic procedures. Threshold to abort right radial access and transition to left radial artery or femoral artery should be low if percutaneous coronary intervention procedure needs to be performed in these patients.
